# Endoscopic ultrasound-guided transgastric drainage of pancreaticopleural fistulas

**DOI:** 10.1055/a-2823-8105

**Published:** 2026-03-19

**Authors:** Katarzyna M. Pawlak, Mateusz Jagielski, Kareem Khalaf, Jacek Piątkowski, Jacek Szeliga, Marek Jackowski

**Affiliations:** 1 49577 Department of General, Gastroenterological and Oncological Surgery, Collegium Medicum, Nicolaus Copernicus University, Nicolaus Copernicus University in Toruń, Toruń, Poland; 2 10071 The Centre for Therapeutic Endoscopy and Endoscopic Oncology and Division of Gastroenterology, St Michael's Hospital, Toronto, Canada; 3 10071 Division of Gastroenterology, St Michael's Hospital, Toronto, Canada

**Keywords:** Pancreatobiliary (ERCP/PTCD), ERC topics, Endoscopic ultrasonography, Pancreas, Intervention EUS

## Abstract

**Background and study aims:**

Pancreaticopleural fistula (PPF) is a rare complication of chronic pancreatitis with prevalence of 0.4%. Drainage under endoscopic ultrasound (EUS) guidance may offer an alternative with unassessed efficacy and safety. This case series aimed to initially assess efficacy and safety of EUS-guided transgastric drainage of PPF.

**Patients and methods:**

This was a retrospective case series that analyzed outcomes of EUS-guided drainage in four patients with PPF. The study was carried out at the Department of General, Gastroenterological, and Oncological Surgery, Collegium Medicum, Nicolaus Copernicus University in Torun (Poland), between 2021–2023.

**Results:**

PPF was identified in four patients with chronic pancreatitis. Fistula tracts were arising from the body (2/4;50%), tail (1/4; 25%), and neck (1/4; 25%). Mean fistula diameter and length were 26.25 mm (15–50 mm) and 107.5 (80–150 mm), respectively. Due to inefficiency of conservative treatment and transpapillary drainage in endoscopic retrograde cholangiopancreatography (ERCP), EUS-guided transgastric/transmural drainage of the PPF was performed using metal or plastic stents. Technical and clinical success was achieved in all patients with no adverse events (AEs). Mean hospital stay was 7.75 days (5–12 days), and symptoms resolved in all patients. All stents were removed. There was no recurrence during mean follow-up of 603.5 days (93–1236 days).

**Conclusions:**

EUS-guided transgastric drainage appears to be a promising alternative for refractory PPF, resulting in technical and clinical success with minimal AEs. Long-term follow-up underscores sustained symptom resolution and absence of recurrence, highlighting its potential in managing this challenging complication.

## Introduction

Pancreaticopleural fistula (PPF) is a rare complication of inflammatory diseases of the pancreas, occurring in less than 0.4% of patients with chronic pancreatitis (CP) 1 2 3 . Formation of PPF relates to the main pancreatic duct or pseudocyst rupture into posterior retroperitoneum and further ascending secretion of pancreatic content into the pleural space thorough the esophageal or aortic hiatus tract 1 .

PPF coexists with pleural effusion, which should be distinguished from reactive pleural effusion that occurs with pancreatitis and requires a different approach. In the context of existing connection, the secretion continues, and thus, treatment is mandatory because pleural effusions are mainly large and refractory with a tendency for rapid accumulation, causing lung entrapment and respiratory failure 4 5 6 . Conservative treatment modalities include analogues of somatostatin (octreotide) or parenteral nutrition, and in most cases, are supported by thoracocentesis. However, efficacy of these options was estimated at 30% to 60% 1 7 8 9 . Hence, procedural options such as endoscopy or surgery remain another therapeutic approach.

The endoscopic approach consists of endoscopic retrograde cholangiopancreatography (ERCP) or endoscopic ultrasonography (EUS)-guided rendezvous ERCP. Although the overall clinical success rate for rendezvous technique is not well established, for ERCP with transpapillary drainage, it may be as high as 92.8% 10 . But in the presence of concurrent anatomical alterations caused by the ongoing inflammatory process in the pancreas, transpapillary drainage is vital and the both aforementioned endoscopic procedures may fail, leading to consideration of surgery. However, the associated risks limit patient selection. Direct EUS-guided drainage of PPF is emerging as a viable option for individuals who do not respond to less invasive alternatives, are unsuitable candidates for surgery, or who refuse more invasive approach. Nevertheless, there has been no prior evaluation of direct EUS-guided drainage of PPF efficacy and safety. Therefore, in this series, we aimed to assess clinical and technical success and safety of direct PPF drainage with stent placement under EUS guidance.

## Patients and methods

This was a retrospective case series conducted at a single center, analyzing patients diagnosed with PPF who underwent direct EUS-guided drainage. The study encompassed all patients admitted to the tertiary Endoscopy Center within the Department of General, Gastroenterological, and Oncological Surgery at Collegium Medicum, Nicolaus Copernicus University in Torun (Poland) between 2021 and 2023. Reporting of this study conforms to the CARE checklist.

### Outcomes assessed

The primary aim was to assess clinical success. Technical success and safety were also analyzed. Clinical success was considered as clinical improvement (symptoms resolution) and pleural effusion or fistula tract regression on imaging without recurrence after stent removal during the follow-up period. Technical success was defined as successful creation of fistulo-gastrostomy with stent placement. Presence of adverse events (AEs) related to the procedure, during the hospital stay or follow-up period was evaluated according to AGREE classification 11 .

### Patients

A total of four patients diagnosed with PPF due to partial pancreatic duct disruption in the setting of complicated chronic pancreatitis were included in this study. All fistulas were confirmed through imaging (computed tomography [CT]) and presenting persistent symptoms. Disconnected pancreatic duct syndrome (DPDs) was excluded by magnetic resonance cholangiopancreatography (MRCP). Although there are no specific recommendations or guidelines for managing PPF, the provided treatment aligned with established knowledge and common step-up practices 2 . The primary conservative approach involved a regimen of intravenous octreotide and antibiotics. After failure, more invasive interventions included transthoracic drainage of pleural effusion. If that was not successful, ERCP was performed by an experienced endoscopist.

**Fig. 1:**
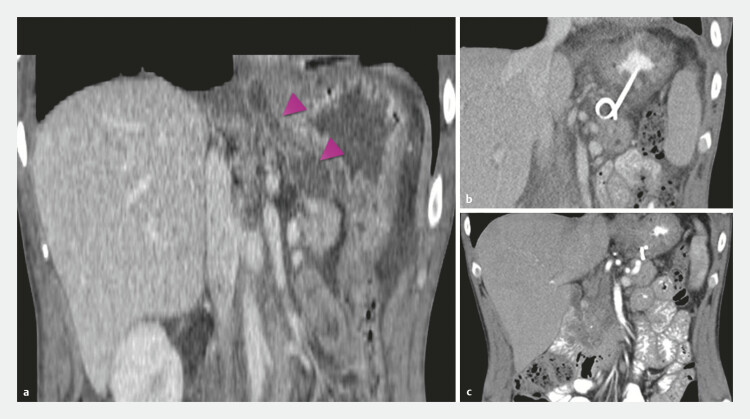
CT images. **a** PPF visualized in CT of abdomen (purple arrow). **b** EUS-guided drainage of PFF with double pigtail stent performed. **c** No visible PFF tract in follow-up CT.

In all cases, transpapillary drainage during ERCPs was attempted at least three times in each case ( Table 1 ). Given lack of transpapillary access in all cases, the EUS-guided rendezvous procedure was not possible. Surgery was considered as a potential treatment option but was ultimately declined by the patients or they were disqualified. Imaging methods comprised chest x-ray to confirm correlation between respiratory symptoms and pleural effusion, followed by chest and abdominal CT for comprehensive evaluation of effusion size, fistula presence, location, and morphology ( Fig. 1 **a** ). Timing of the procedure varied according to patient clinical condition, while allowing for a step-up approach. Because there was no clinical improvement after applying the aforementioned management, EUS-guided drainage was proposed and performed after agreement. Patient characteristics are shown presented in Table 1 **.**

**Table:** **Table 1** Pre-procedure characteristics and initial treatment.

	Case 1	Case 2	Case 3	Case 4
**Demographics**				
Gender	Male	Male	Male	Male
Age (years)	42	43	55	61
**Clinical presentation**				
Dyspnea	Present	Present	Present	Present
Low-grade fever	Present	-	-	-
Fever with chills	-	-	Present	Present
Epigastric pain	Present	-	-	Present
Weight loss (kg)	13	-	-	-
Respiratory failure	-	Present	-	-
Kidney failure	-	Present	-	-
Liver failure	-	Present	-	-
Sepsis	-	Present (SOFA 4)	-	-
**Etiology of PPF**	CP	CP	CP	CP
**Imaging**				
CT general findings	Fluid in left pleural cavity	Fluid in left pleural cavity	Fluid in right pleural cavity	Fluid in left pleural cavity
	Features of CP	Features of CP	Features of CP	Features of CP
MRCP	No evidence of DPDS	No evidence of DPDS	No evidence of DPDS	No evidence of DPDS
Fistula measurements (diameter x length; mm)	15 × 80	20 × 100	50 × 150	20 × 100
Fistula distance from gastric wall (mm)	Directly adjusted to gastric wall	10	25	15
Site of connection to MPD	Pancreatic tail	Pancreatic body	Pancreatic neck	Pancreatic body
X-ray	Fluid in left pleural cavity	Fluid in left pleural cavity	Fluid in right pleural cavity	Fluid in left pleural cavity
	Calcifying CP	Calcifying CP	Calcifying CP	Calcifying CP
**Initial interventions**				
Octreotide	Yes	Yes	Yes	Yes
Transthoracic drainage	Yes	Yes	Yes	Yes
Reason for failed cannulation during ERCP	PD stenosis in head of pancreas (3 ERCP attempts)	Infiltration of periampullary area due to CP exacerbation	Inflammatory tumor of head of pancreas (3 ERCP attempts)	Infiltration of periampullary area due to CP exacerbation
CP, chronic pancreatitis; DPDS, disconnected pancreatic duct syndrome; ERCP, endoscopic retrograde pancreatography; MPD, main pancreatic duct.				

### EUS procedure

All EUS-guided drainage procedures were performed by one endoscopist (Mateusz Jagielski), under general anesthesia and with endotracheal intubation.

The approach for EUS-guided drainage was primarily based on imaging studies, including CT and MRCP and was developed after consultation with the radiologist. Once a potential site for fistula localization from the stomach was identified, diagnostic EUS was performed as part of the EUS-guided procedure. The procedure was performed using a linear therapeutic EUS scope and began with endosonographic assessment. EUS-guided pancreaticogastrostomy was declined due to anatomical circumstances which could potentially lead to procedure failure and unnecessary complications. Then further evaluation was performed to visualize the fistula and to determine the distance between gastrointestinal tract wall and lumen of the fistula. The fistulous tract was seen from the posterior wall of the stomach, appearing as a hypoechoic, tubular structure with no Doppler enhancement. Given that no other anatomical structures in the vicinity correlated with the CT findings, the fistula was confirmed.

**Fig. 2:**
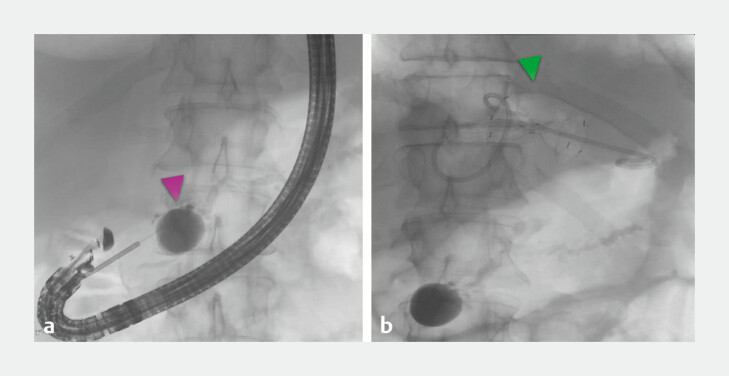
X-ray images. **a** Fistula tract imitating pseudocyst filled with contrast, further tract not visible due to fast contrast washout. **b** EUS-guided transgastric drainage of PFF with biflanged metal stent placement and double pigtail stent placement.

In all cases, the distance was less than 25 mm, enabling transgastric access. First, the fistula lumen was targeted with a 19G fine-needle aspiration needle with subsequent liquid content aspiration to exclude blood presence and obtain material for laboratory analysis (cytology, cultures, amylase, carcinoembryonic antigen, glucose level). That was followed by contrast injection for fistulography and descending pancreatography performance under the fluoroscopy to assess fistula tract direction and anatomy and visualize the connection between the main pancreatic duct (MPD) and pleural effusion and to also exclude DPDS ( Fig. 2 **a** ). Then, the standard 0.035-inch wire was advanced through the needle, which was exchanged for a 10F cystotome for fistulo-gastrostomy tract creation using diathermy settings. Of note, for all procedures, a 10F system was used, which allowed advancement of stents without need for multiple device exchanges, which, in a potentially narrow space compared with a large pseudocyst, may increase risk of guidewire dislodgement and, consequently, procedure-related complications or failure. Hence the tract was not dilated and diameter of 10F was enough to insert a collapsed stent. Finally, plastic stents or biflanged metal stents (BFMSs) were introduced in a similar fashion as for pseudocyst drainage, under endoscopic, fluoroscopic and partially endosonographic guidance, achieving the desired position and visible drainage ( Fig. 1 **b, Fig. 2 b** ). Stent selection was left to the discretion of the endoscopists, but distance between the gastric wall and fistula lumen and fistula diameter were taken into consideration as decision-making factors, and hence, for with a larger distance, to minimize risk for migration, a BFMS was selected. In patients with the worst clinical manifestation, a nasogastric drain was inserted along the plastic double pigtail stent (DPS) and active drainage was provided for 7 days. Of note, the transthoracic drain was kept in until EUS-guided drainage was performed to monitor fistula output.

### Post-procedure management

All patients received antibiotics and supportive treatment, including pain management and nutritional therapy. Of note, octreotide was not continued as supportive treatment after drainage. Transthoracic drainage was removed when output from fistulas was less than 50 mL a day and clinical improvement was observed.

### Follow-up

The first follow-up assessment was conducted 4 weeks after the index discharge. At that time, a chest and abdomen CT was performed to assess degree of advancement of the fistula and pleural effusion ( Fig. 1 **c** ). The stent was removed when there was complete regression of pleural effusion and fistula, and exchanged to plastic when one of those two conditions persisted. Thereafter, regardless of stent presence, patients were evaluated clinically and with CT during consecutive control visits. The intervals for control visits were determined by the endoscopist. Follow-up continued despite lack of recurrence in outpatient settings for further assessment of patient clinical status.

### Statistical analysis

Descriptive statistical analysis was conducted to characterize and summarize key features of the dataset.

## Results

Four patients (all male; mean age 50.25 years [42-61]) with chronic pancreatitis with diagnosed PPF were qualified for EUS-guided drainage. Patient characteristics are presented in Table 1 .

### Pre-procedure management

Before EUS-guided drainage, all patients underwent the same step-up approach. That included classic transthoracic drainage of the pleural effusion, which failed in all cases, resulting in recurrence of pleural effusion and worsening of symptoms. Simultaneously, all patients underwent at least three ERCPs. However, all attempts failed due anatomical difficulties including severe stenosis of the MPD ( Table 1 ).

### Characteristics of PPF

Chest and abdomen CT facilitated determination of morphological features of fistula and pleural effusions. Fistula tracts arise from various parts of the pancreas including the body (2/4;50%), tail (1/4; 25%), and neck (1/4; 25%). Mean fistula diameter and length were 26.25 mm (15–50 mm) and 107.5 (80–150 mm), respectively. Mean distance between the fistula tract and gastric wall was 12.5 mm (15–25 mm). Mean pleural effusion size was 107.5 mm (80–150 mm).

### EUS-guided procedure

EUS-guided drainage of the PPF was performed via transgastric access in all patients, from two locations: below the gastric cardia (2/4; 50%) and gastric body (2/4; 50%). DPss (7F, 9–12 cm) were placed in three patients and BFMSs (30 × 16 mm) in one, with a DPS through the stent lumen. Technical and clinical success was achieved in all patients (4/4;100%). Mean procedure time was 33 minutes (26–47 min). There were no AEs related to the procedure during the hospital stay or follow-up period. Procedure characteristics are presented in Table 2 .

**Table:** **Table 2** Procedure characteristics.

	Case 1	Case 2	Case 3	Case 4
**Procedure data**				
Sedation	Endotracheal intubation	Endotracheal intubation	Endotracheal intubation	Endotracheal intubation
Access site of transmural drainage	Below the gastric cardia	Below the gastric cardia	From the gastric body	From the gastric body
Needle type	19G FNA	19G FNA	19G FNA	19G FNA
Wire type	0.025-inch	0.035-inch	0.035-inch	0.035-inch
Dilatator type	Cystotome 10F	Cystotome 10F	Cystotome 10F	Cystotome 10F
Metal stent type, size	-	-	Biflanged metal stent 30 mm x 16 mm	-
DP type, size	Two DP stents 7F x 9 cm 7F x 12 cm	DP stent 7F x 9 cm Nasal tube 7F	DP stent through lumen of SEMS 7F x 12 cm	DP stent 7F x 12 cm
Procedure time (min)	47	31	26	28
Adverse events	None	None	None	None
DP, double pigtail; FNA, fine-needle aspiration; SEMS, self-expandable metal stent.				

### Post-procedure management

All patients were continued on antibiotics, supportive pain management, and nutritional treatment. Mean post-procedure hospital stay was 7.75 days (5–12 days) and symptoms resolved in all patients.

### Follow-up

Because there was no DPDS, stents were removed when complete fistula tract regression was confirmed on CT scan. In two patients, plastic stents were removed after 6 months, but in one patient, the plastic stent was exchanged after 2 months due to incomplete resolution and subsequently removed after another 2 months when resolution was observed. In the patient with a metal stent, exchange to a plastic stent was performed after 4 weeks, which as removed 3 months later. Mean follow-up was 903.5 days (451–1536 days). All patients (4/4; 100%) achieved clinical success with collection resolution and fistula tract closure. No recurrence was observed. Despite completed endoscopic treatment, the patients remain under outpatient supervision pending complex management for chronic pancreatitis. Follow-up characteristics are presented in Table 3 .

**Table:** **Table 3** Follow-up characteristics.

	Case 1	Case 2	Case 3	Case 4
**Follow-up data**				
Total time of follow-up (days)	1536	1234	451	393
Follow-up	Stent removal after 6 months	Stent removal after 6 months	BFMS removal after 1 month with replacement to 7F x 9 cm Stent removal after 3 months	Stent replacement to DP stent 7F x 9 cm after 2 months Stent removal after 2 months
Clinical success	Achieved	Achieved	Achieved	Achieved
BFMS, biflanged metal stent; DP, double pigtail.				

## Discussion

**Fig. 3:**
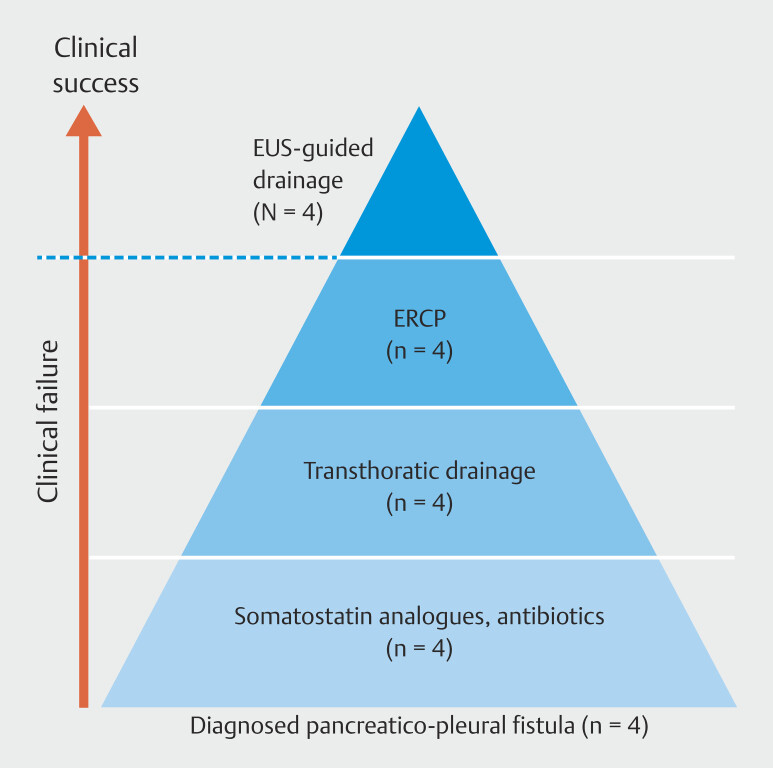
Step-up approach to treatment of pancreatico-pleural fistulas.

There are no explicit guidelines for management of PPFs. Based on the current literature, treatment it mainly focuses on a step-up approach ( Fig. 3 ), beginning with conservative options such as octreotide, progressing to invasive methods such as thoracocentesis, and endoscopic solutions including ERCP with quite high success rates reaching up to 96%, when transpapillary access is possible 10 . Surgical intervention, with a success rate of 80% to 90%, is the next step after less invasive methods fail, but is limited by higher risk of AEs 10 . Hence, determining the treatment strategy is challenging at various levels, especially when less invasive methods fail, and surgery is restricted by multiple factors, resulting in a lack of optimal treatment modality for this patient group.

In our cohort, four male patients with CP and diagnosed PPF underwent EUS-guided transgastric drainage. There are no publications to date on EUS-guided drainage of PPF performance or assessment in this setting. Hence, lack of published data regarding EUS-guided drainage of PPF makes comparison impossible, but at least outcomes of this study are consistent with results reported for other types of EUS-guided drainage, confirming general benefits of the therapeutic modality, including safety and technical accessibility in expert hands 12 .

The procedure was selected as a less invasive option than surgery when previous attempts at classic transthoracic drainage and ERCP failed. The patients also had not consented to surgery. The EUS-guided intervention was enabled by unique visualization of a wide fistula tract, which in most cases is typically narrow and difficult to localize. Hence, transgastric drainage was feasible by using DPSs or BMSs and resolution of symptoms and pleural effusions was achieved in all cases, with relatively short, mean procedure time of 33 minutes. Stent removal was determined by the lack of existing DPDS, which could be mirrored by clinical success in all cases and no fistulas recurrence was observed during the follow-up.

Endotherapy has proven to be an effective treatment modality for managing disruptions and leaks in MPD 1 2 3 4 5 6 . Partial MPD disruption occurs more frequently than complete duct disruption 6 . Evidence suggests that endotherapy is particularly effective in treatment of partial MPD disruption compared with complete disruption, particularly in patients with pancreatitis 6 . Jang et al. demonstrated significantly better therapeutic outcomes in patients with partial MPD disruption compared with those with complete duct disruption 6 . In addition, the study reported a higher incidence of recurrent post-inflammatory fluid collections and recurrence of pancreatitis in patients with complete duct disruption following initial treatment, compared with those with partial disruption 6 . Similar conclusions were drawn by Shrode et al., who found that stenting MPD during ERCP for post-inflammatory fluid collections yields positive therapeutic results only in patients with partial duct disruption. In contrast, in patients with complete duct disruption, insertion of a prosthesis did not provide any significant therapeutic benefit 6 . In early reports on treatment of disconnected duct syndrome, Deviere et al. concluded that endotherapy is both effective and safe, although they noted a high rate of recurrence of post-inflammatory fluid collections in the region of MPD disruption 13 . Furthermore, DPDS has been shown to worsen outcomes of endotherapy in patients with MPD disruption 5 7 8 9 . In the present study, all cases involved partial MPD disruption, and no cases of DPDS were diagnosed, a factor that likely contributed to favorable outcomes observed with endoscopic treatment.

Assessment of our outcomes remains impossible due to a lack of data; however, given slight similarity to pseudocyst management, a comparison with that may be relevant. Yang et al. 14 showed that transmural drainage of pancreatic pseudocysts without transpapillary drainage is sufficient for regression of post-inflammatory pancreatic fluid collection. In our study EUS-guided transmural drainage of PPF was performed when transpapillary access failed and outcomes appeared to be encouraging. Although PPF is clinically different than pseudocysts and frequently involves severe respiratory symptoms that require extended time for recovery, the outcome of EUS-guided transgastric treatment is similar. Namely, drainage of PPF decreases PD pressure and helps to rapidly close the PD disruption. Hence, in all four of our cases, the PPF communicating with the MPD regressed after transmural drainage, providing evacuation of serous content from the fistula to achieve complete regression of PPF. Given this observation, we believe that patients with PPF and PD disruption should undergo transpapillary drainage or transmural drainage. A single means of access (transmural or transpapillary) seems sufficient to close the PPF. On the other hand, dual-modality drainage of PPF (transmural and transpapillary) does not seem to be a good treatment option because it may negatively affect long-term resolution of PPF. Further to the comparison to EUS-guided drainage, length of hospital stay was comparable to studies on pseudocyst EUS-guided drainage 15 . Moreover, absence of AEs during the hospital stay and follow-up period further underscores efficacy and safety of EUS-guided drainage, positioning it as a promising treatment modality for PPF, especially when conventional, minimally invasive approaches prove unsuccessful.

With regard to technical considerations and stent selection, an approach analogous to that used for pseudocyst drainage may be applicable; however, anatomical factors should always be taken into account, particularly distance between the gastric wall and the fistulous tract. Based on our clinical experience 16 , use of long BFMSs may be considered to reduce risk of stent migration.

Of note, lack of recurrence following complete stent removal was observed. Absence of recurrence over a prolonged follow-up period (451–1,536 days) may be hypothesized to reflect spontaneous resolution of pancreatic ductal stenosis as the result of decompression of the MPD and associated inflammatory changes over time and/or persistence of a mature tract at the site of access, which may have facilitated sustained decompression and, thereby, reduced risk of recurrence.

Major limitations of the study include the small sample size, retrospective, observational nature, and short follow-up for one case; however, all patients continue to be followed up in outpatient settings On the other hand, PPF as complication of CP is extremely rare, accounting for 0.4% prevalence in literature. Because the clinical manifestation may be catastrophic, establishing further step-up management and adjusting all possible minimally invasive alternatives for different scenarios is needed.

More robust data from multicenter, prospective studies are essential for confirming these initial findings. However, this pilot series is the first to assess direct EUS-guided drainage of PPF.

Nevertheless, the promising outcomes from our study contribute greatly to the current state of knowledge about endoscopic treatment of PPF because consensus currently is lacking regarding optimal management of patients with PPFs in the course of pancreatitis. Despite this, EUS-guided drainage of PPF requires further studies conducted on larger groups of patients. Recognizing the complexity of open surgery for pancreatitis, there is a crucial need to explore less invasive approaches for addressing complications associated with this condition.

## Conclusions

In conclusion, EUS-guided transgastric drainage may be a safe and effective alternative for managing patients with PPF refractory to conventional interventions, who are not suitable for surgery, and in whom transpapillary access has failed, demonstrating both technical and clinical success with limited AEs in this case series.
